# Publisher Correction: Translation efficiency driven by CNOT3 subunit of the CCR4-NOT complex promotes leukemogenesis

**DOI:** 10.1038/s41467-024-53071-1

**Published:** 2024-10-07

**Authors:** Maryam Ghashghaei, Yilin Liu, James Ettles, Giuseppe Bombaci, Niveditha Ramkumar, Zongmin Liu, Leo Escano, Sandra Spencer Miko, Yerin Kim, Joseph A. Waldron, Kim Do, Kyle MacPherson, Katie A. Yuen, Thilelli Taibi, Marty Yue, Aaremish Arsalan, Zhen Jin, Glenn Edin, Aly Karsan, Gregg B. Morin, Florian Kuchenbauer, Fabiana Perna, Martin Bushell, Ly P. Vu

**Affiliations:** 1 Faculty of Pharmaceutical Sciences, University of British Columbia, Vancouver, Canada; 2 Terry Fox Laboratory, British Columbia Cancer Research Centre Vancouver, Vancouver, Canada; 3 Department of Experimental Medicine, University of British Columbia, Vancouver, Canada; 4 CRUK Beatson Institute, Glasgow, UK; 5 School of Cancer Sciences, University of Glasgow, Glasgow, UK; 6 Department of Medicine, Indiana University Simon Comprehensive Cancer Center, Indianapolis, IN USA; 7 Genome Sciences Centre, British Columbia Cancer Research Centre, Vancouver, Canada; 8 Bioinformatics program, University of British Columbia, Vancouver, Canada; 9 Memorial Sloan Kettering Cancer Center, New York, NY USA; 10 Department of Medical Genetics, University of British Columbia, Vancouver, Canada; 11 Department of Blood and Marrow Transplant and Cellular Immunotherapy, Moffit Cancer Center, Tampa, FL USA

**Keywords:** Acute myeloid leukaemia, Translation, RNA decay

Correction to: *Nature Communications* 10.1038/s41467-024-46665-2, published online 15 March 2024

In this article the ‘*The authors contributed equally’ statement was omitted from the paper. The original article has been corrected.

