# Supplementary material for: Translation efficiency driven by CNOT3 subunit of the CCR4-NOT complex promotes leukemogenesis
Source: Nat Commun. 2024 Mar 15;15:2340. doi: 10.1038/s41467-024-46665-2 (PMC10943099; doi:10.1038/s41467-024-46665-2)
Supplement: Supplementary file 1 — Supplementary information [file 41467_2024_46665_MOESM1_ESM.pdf]

Supplementary Fig. 1

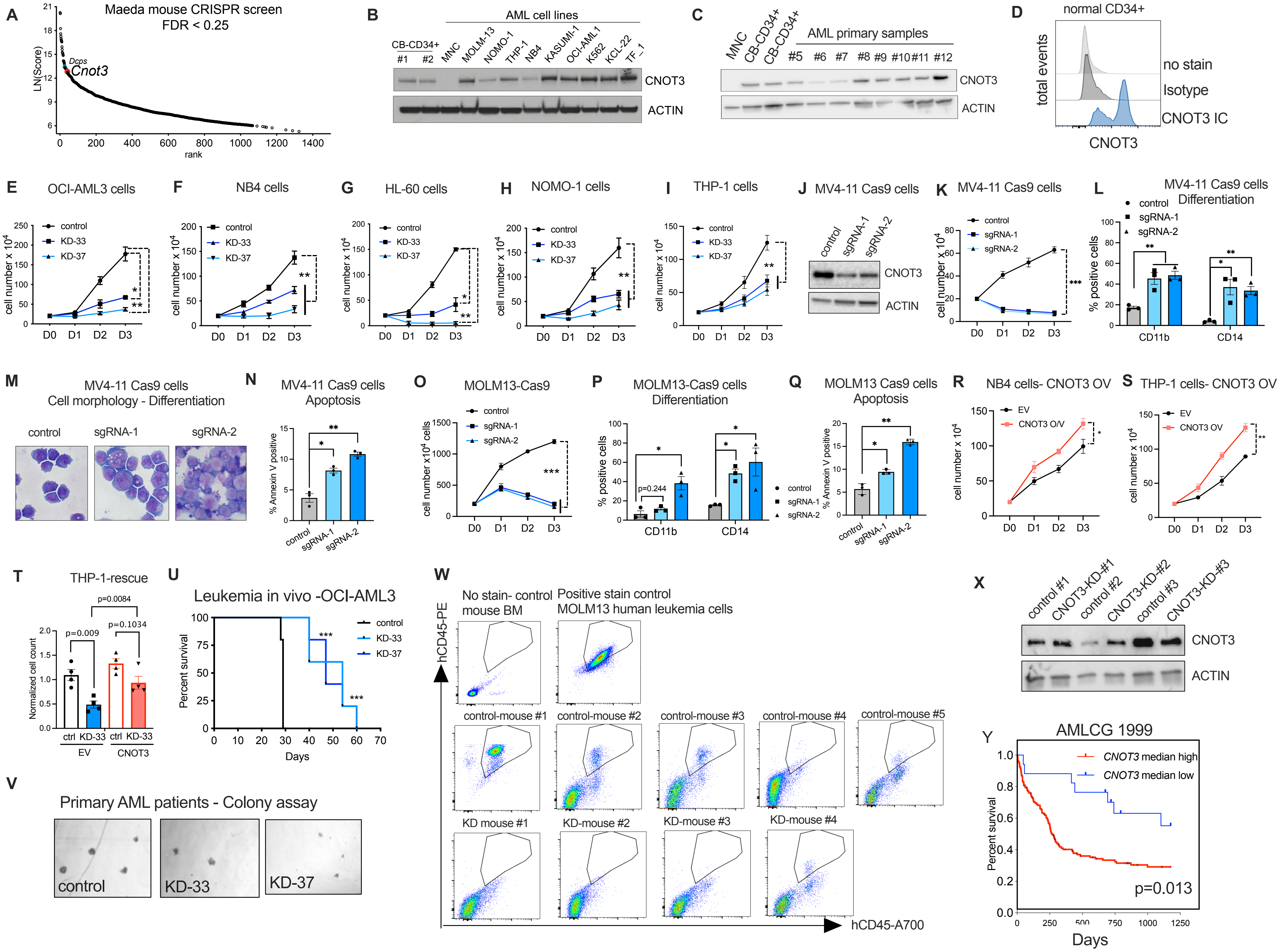

Supplementary Fig. 2.

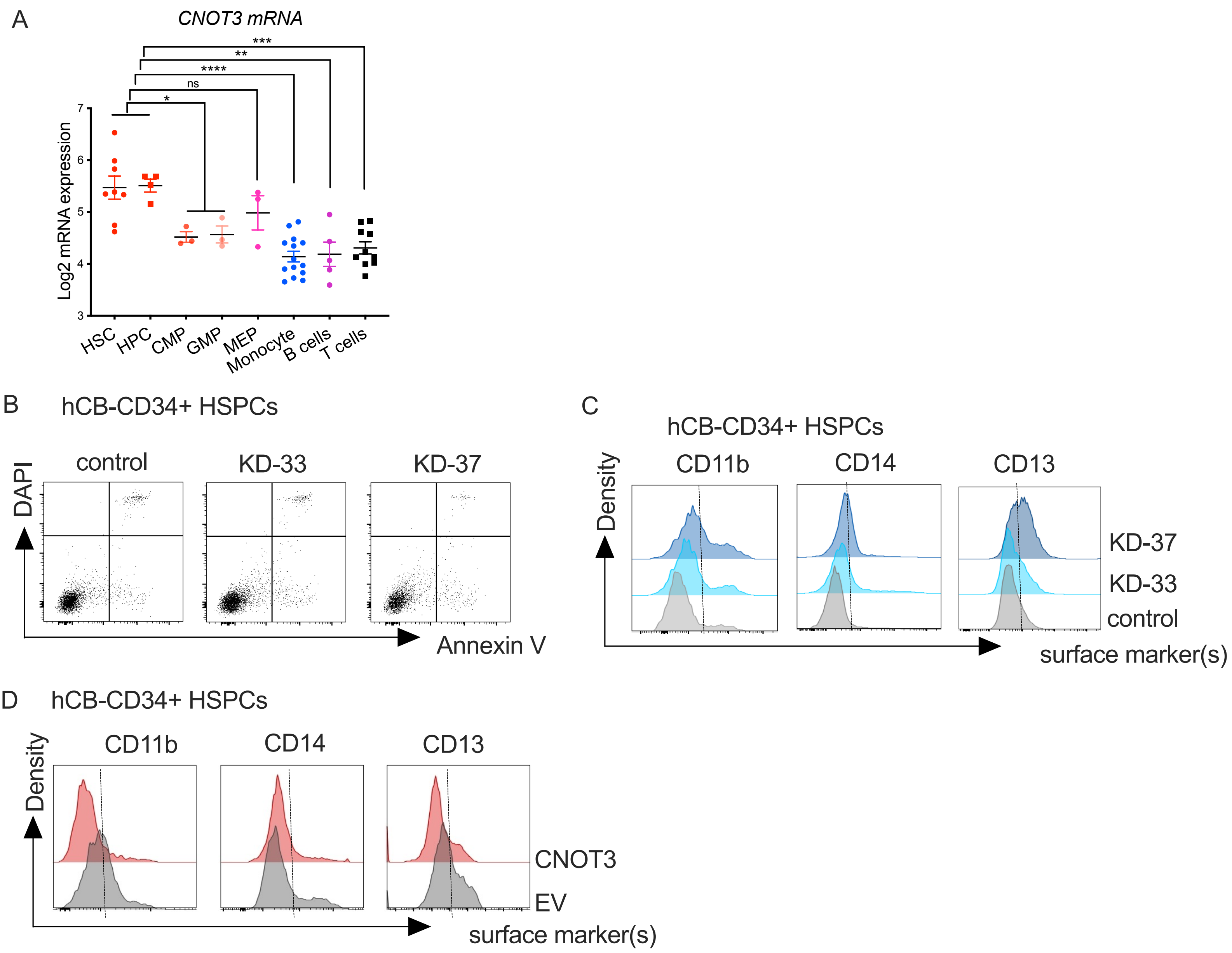

Supplementary Fig.3.

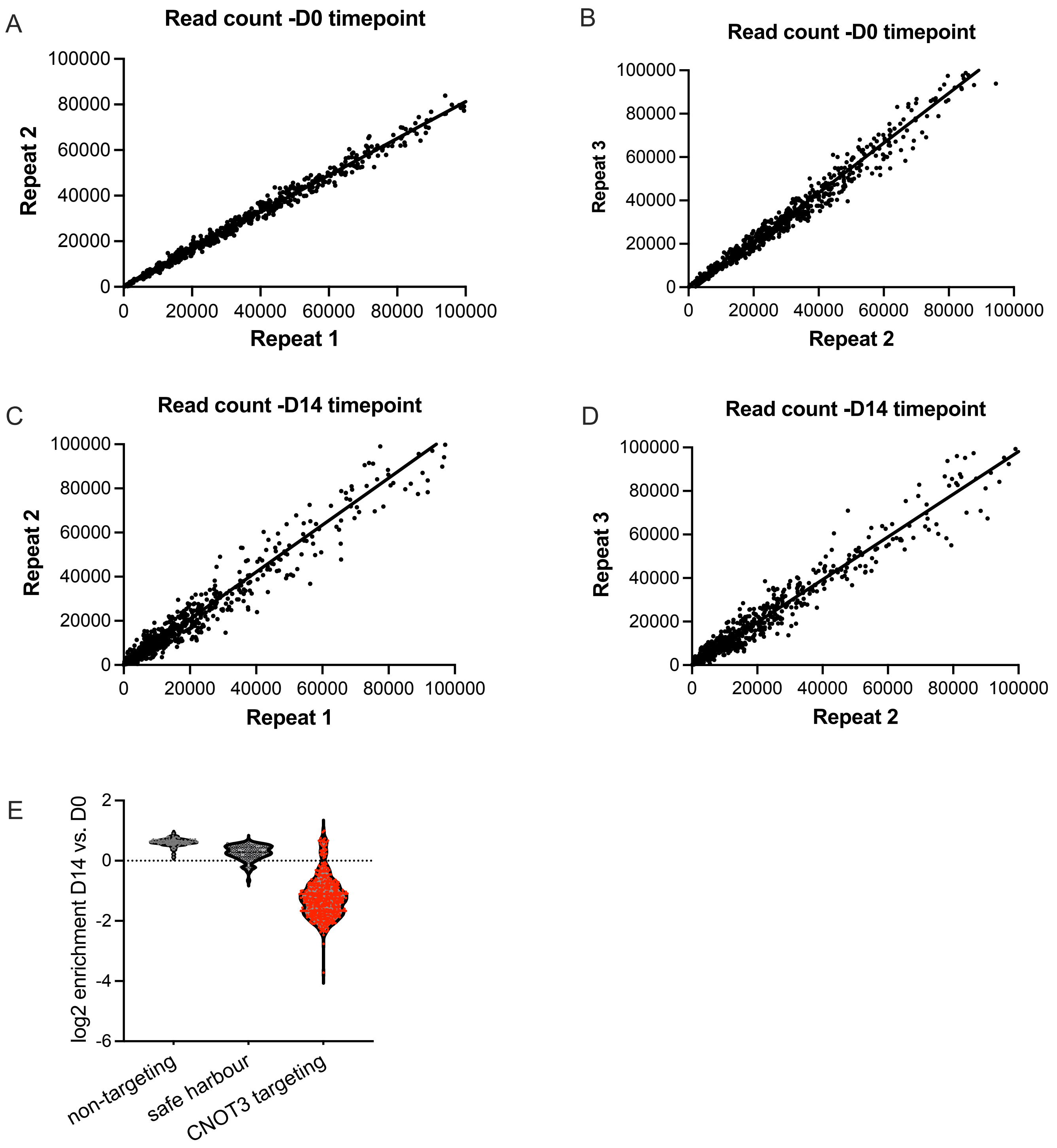

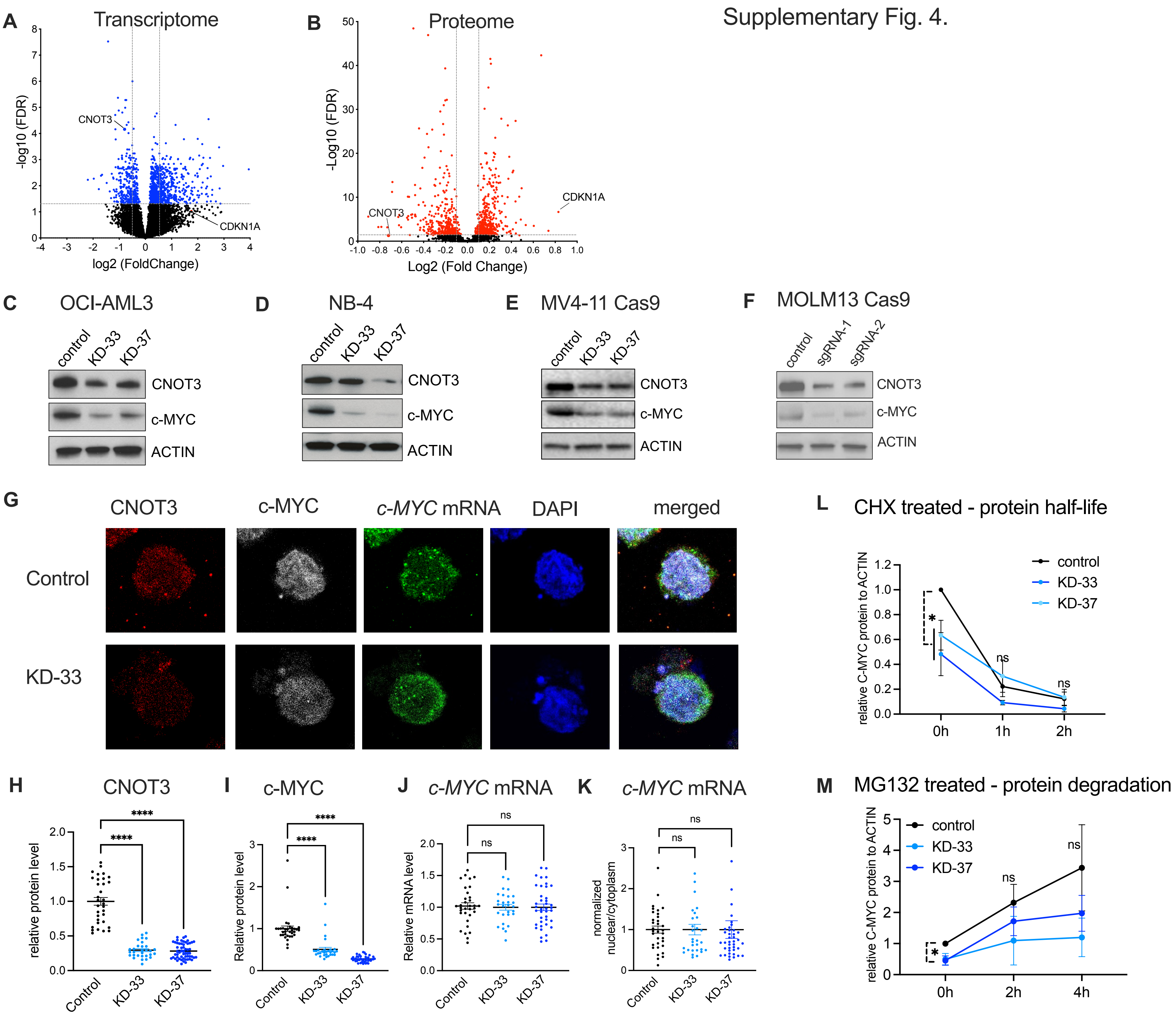
Supplementary Fig. 4.
A
Transcriptome
-log10 (FDR)
log2 (FoldChange)
CNOT3
CDKN1A
B
Proteome
-Log10 (FDR)
Log2 (Fold Change)
CNOT3
CDKN1A
C
OCI-AML3
control
KD-33
KD-37
CNOT3
c-MYC
ACTIN
D
NB-4
control
KD-33
KD-37
CNOT3
c-MYC
ACTIN
E
MV4-11 Cas9
control
KD-33
KD-37
CNOT3
c-MYC
ACTIN
F
MOLM13 Cas9
control
sgRNA-1
sgRNA-2
CNOT3
c-MYC
ACTIN
G
CNOT3
c-MYC
c-MYC mRNA
DAPI
merged
Control
KD-33
L
CHX treated - protein half-life
relative C-MYC protein to ACTIN
control
KD-33
KD-37
0h
1h
2h
ns
ns
H
CNOT3
relative protein level
Control
KD-33
KD-37
I
c-MYC
Relative protein level
Control
KD-33
KD-37
J
c-MYC mRNA
Relative mRNA level
Control
KD-33
KD-37
ns
ns
K
c-MYC mRNA
normalized nuclear/cytoplasm
Control
KD-33
KD-37
ns
ns
M
MG132 treated - protein degradation
relative C-MYC protein to ACTIN
control
KD-33
KD-37
0h
2h
4h
ns
ns

Supplementary Fig.5.

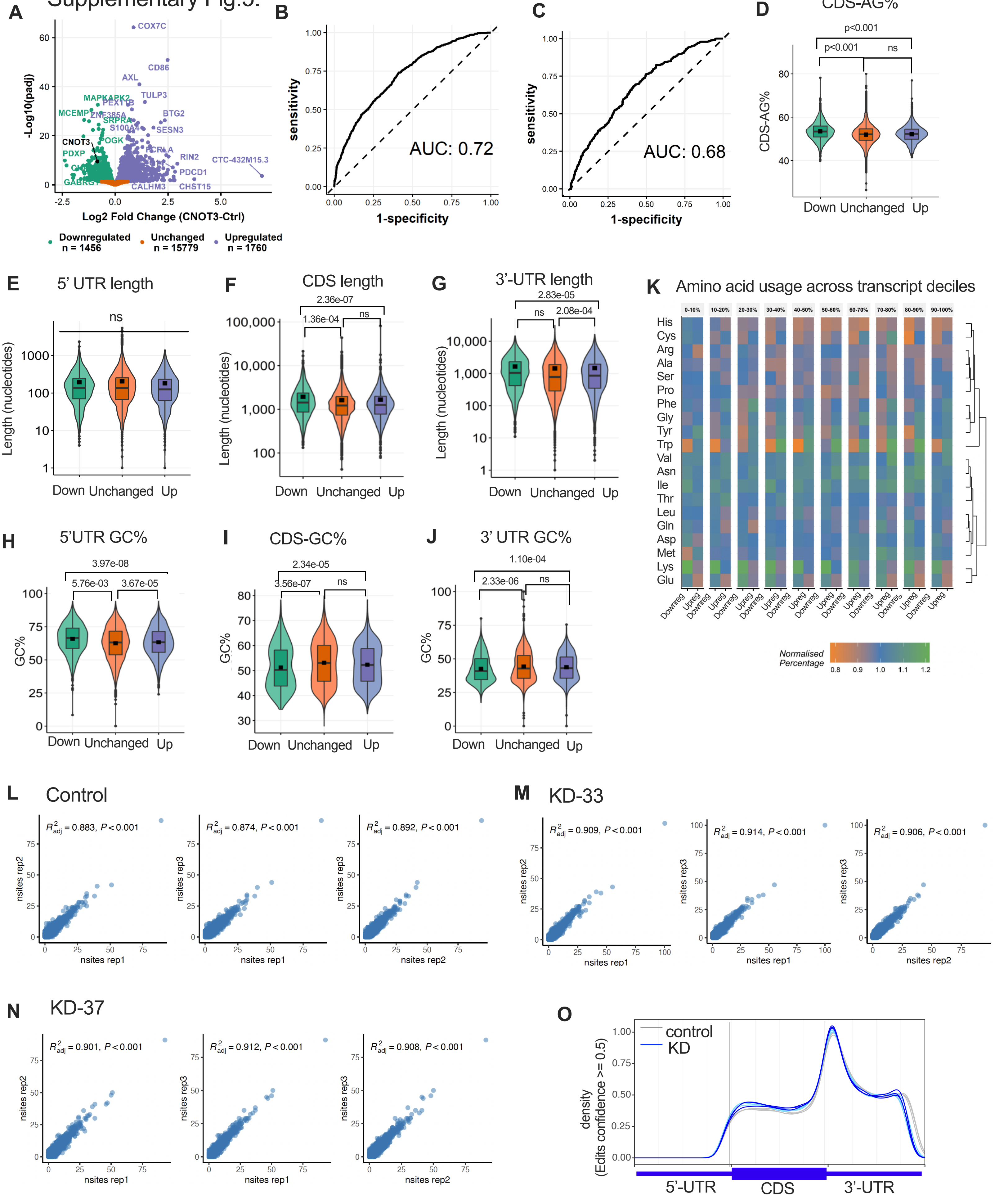

Supplementary Fig. 6

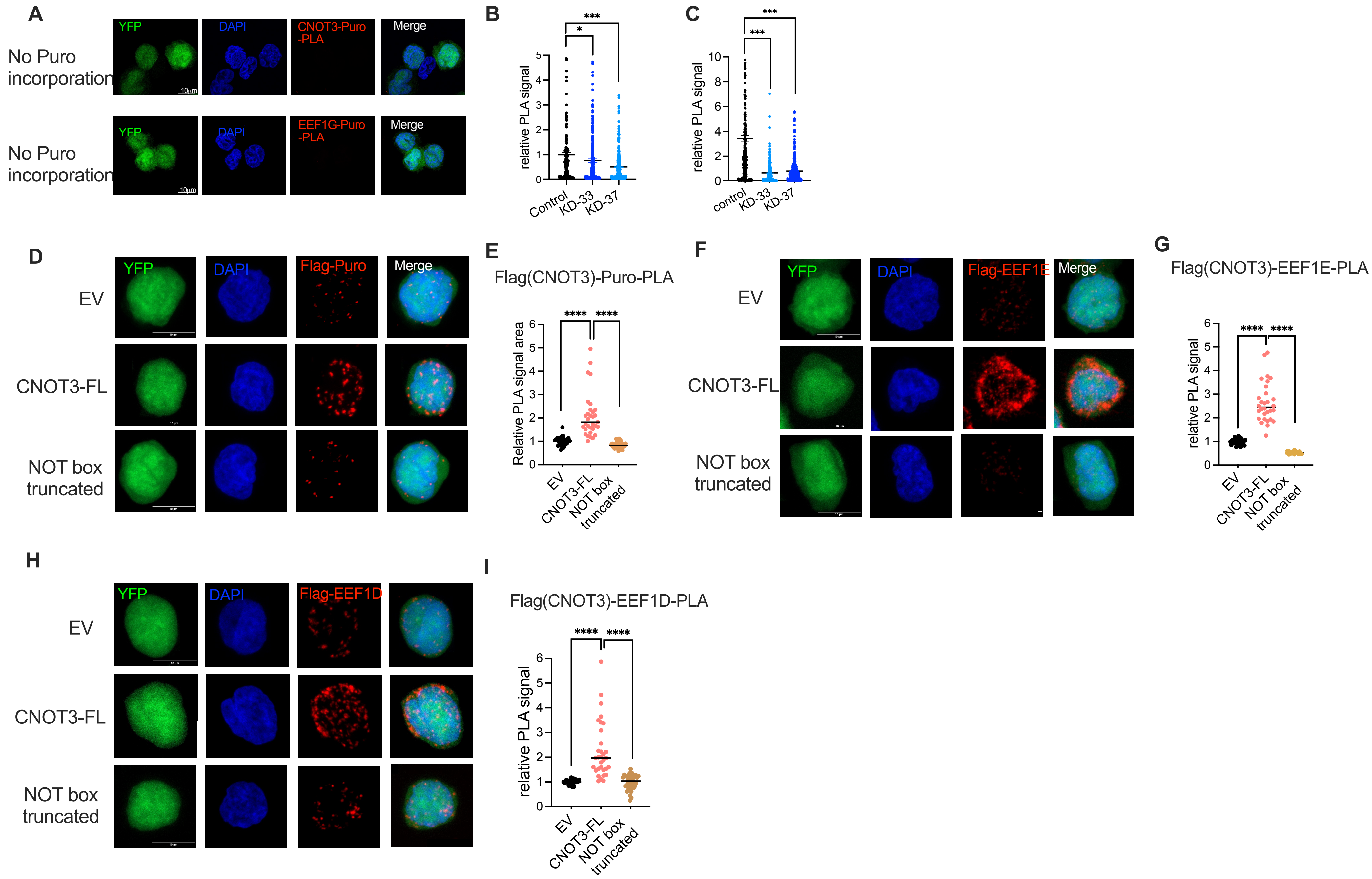

Supplementary figure legends

Supplementary figure 1. CNOT3 promotes survival and growth of leukemia cells

(**A**) CRISPR score rank for essentiality for cell survival of CNOT3 in the Maeda mouse genome wide screen for genes essential for leukemia. (**B**) Immunoblots for CNOT3 protein and ACTIN (loading control) in normal cord blood (CB) derived CD34+ HSPCs and AML cell lines. (**C**) Immunoblots for CNOT3 protein and ACTIN (loading control) in CB-CD34+ cells and in primary AML patient cells vs. mononuclear cells (MNC). (**D**) Representative flow plots showing results from intracellular staining of CNOT3. (**E-I**) A panel of AML cell lines with diverse genetic backgrounds were transduced with shRNA control or *CNOT3* KD and cell proliferation was evaluated: OCI-AML3, NB4, HL-60, NOMO-1 and THP-1. Cells were selected for puromycin resistance and assayed 3 days post-transduction. n = 3 independent experiments, * $p < 0.05$, ** $p < 0.01$, two-tailed Student's t test. All graphs show data as mean+/- s.e.m. (**J-N**) MV4-11 Cas9 cells were transduced with lentiviruses expressing either an empty vector (control) or CNOT3-targeting sgRNAs (sgRNA-1 and sgRNA-2). Cells were selected based on GFP positivity and assayed 3 days post-transduction. n = 3 independent experiments, * $p < 0.05$, ** $p < 0.01$, two-tailed Student's t test. (**J**) Immunoblots showing efficient ablation of CNOT3. ACTIN serves as loading control. (**K**) Cell proliferation. (**L**) Quantitative summary of flow cytometry analysis of myeloid differentiation markers CD11b and CD14. (**M**) Representative H&E images of morphological evaluation cells upon CNOT3 depletion. (**N**) Percentage of apoptotic cells by flow cytometry analysis for Annexin-V positivity. (**O-Q**) MOLM13 – Cas9 cells were transduced with lentiviruses expressing either an empty vector (control) or CNOT3-targeting sgRNAs (sgRNA-1 and sgRNA-2). Cells were selected based on GFP positivity and assayed 3 days post-transduction. n = 3 independent experiments, * $p < 0.05$, ** $p < 0.01$, two-tailed Student's t test. (**O**) Cell proliferation. (**P**) Quantitative summary of flow cytometry analysis of myeloid differentiation markers CD11b and CD14. (**Q**) Percentage of apoptotic cells by flow cytometry analysis for Annexin-V positivity. (**R-S**) Cell proliferation of NB4 and THP-1 cells transduced with lentiviruses expressing empty vector (control) or cDNA expressing CNOT3 (CNOT3-OV). Transduced cells were sorted based on YFP positivity 3 days post transduction. n = 3 independent experiments, ** $p < 0.01$, two-tailed Student's t test. (**T**) Cell growth at day 3 after plating of THP-1 cells transduced lentiviruses expressing empty vector (control) or cDNA expressing CNOT3 (CNOT3-OV) and either control or CNOT3 KD-33 shRNA. Cells were selected based on YFP positivity and puromycin resistance. n = 4 independent experiments, ** $p < 0.01$, two-tailed Student's t test. (**U**) Kaplan–Meier curves showing survival of three groups of mice injected with OCI-AML3 cells

transduced with either control vs. KD-33 and KD-37. Control n= 5; KD-33 n=5; KD-37 n=5. p values were calculated using Log-rank (Mantel-Cox) test. ***p<0.001. (**V**) Representative images showing colonies formed by primary AML cells transduced with control vs. KD-33 or KD-37. (**W**) Representative flow plots showing gating of population of human MOLM13 leukemia CD45+ cells in recipient animals. (**X**) Immunoblots for CNOT3 protein and ACTIN (loading control) in leukemia cells outgrown in mice succumbed to leukemia, showing re-detection of CNOT3 in previously knocked down samples. ACTIN serves as loading control. (**Y**) High expression of *CNOT3* mRNA correlates with poor prognosis in AML patients in three independent cohorts. Kaplan–Meier curves showing outcomes of AML patients in German AMLCG 1999 trial high n=146 vs. low n=17.

Supplementary figure 2. CNOT3 suppress differentiation of primary HSPCs

(**A**) *CNOT3* mRNA expression across different cell types in human hematopoietic system. Data obtained from the Bloodspot database https://servers.binf.ku.dk/ for *CNOT3* probe 229143_at. Hematopoietic stem cells (HSC) n=8; Hematopoietic progenitor cells (HPC) n=4; Common Myeloid Progenitor (CMP) n=3; Granulocyte Monocyte Progenitor (GMP) n= 3; Megakaryocyte Erythrocyte Progenitor (MEP) n=3; Monocyte n=14; B cells n=5; T cells n= 10. ns not significant, * p<0.05, **p < 0.01, *** p<0.001 two-tailed Student's t test. (**B-C-D**) Representative flow plots showing Annexin V and DAPI staining for apoptotic analysis and CD11b, CD14 and CD13 for myeloid differentiation.

Supplementary figure 3. NOT box domain is essential for CNOT3 function in AML

(**A-D**) Scatter plots showing read counts obtained from each biological repeat (i.e., three independent experiments using three batches of cells for independent viral transduction and selection) from CRISPR domain screening at both time points D0 and D14, showing high reproducible results from replicates. (**E**) Log2 enrichment of on-target sgRNAs vs. control sgRNAs which are either non-targeting sgRNAs or sgRNAs mapped to safe-harbour regions of the genome.

Supplementary figure 4. CNOT3 controls translation of c-MYC

(**A-B**) Volcano plots showing all profiled genes in cells transduced with lentiviruses carrying CNOT3-KD vs. control shRNAs (**A**) RNA-seq analysis and (**B**) Proteomic analysis. Genes with FDR ≤ 0.05 are highlighted. (**C-F**) Immunoblots for CNOT3, c-MYC and ACTIN (as loading control) expression in (**G**) OCI-AML3, NB4, MV4-11 Cas9 and MOLM13 Cas cells. (**G**) Representative immunofluorescent (IF) images of CNOT3, c-MYC proteins and *c-MYC* mRNA in

MOLM13 cells upon CNOT3 depletion. (**H-K**) Quantitative summary of IF results for (**H**) CNOT3, (**I**) c-MYC protein, (**J**) *c-MYC* mRNA and (**K**) abundance of *c-MYC* mRNA in nucleus vs. cytoplasm. Each dot represent data obtained from one cell. All graphs show data as mean+/- s.e.m. Two-tailed Student's t test. ns no significant * p< 0.05, **p<0.01, *** p<0.001. (**L-M**) Quantitative summary of c-MYC levels over time after treatment of cells with (**L**) cycloheximide (CHX) and (**M**) proteasome inhibitor MG132 to assay for protein half-life and degradation upon CNOT3 depletion. ACTIN serves as control. All graphs show data as mean+/- s.e.m, n=3 independent experiments. Two-tailed Student's t test. ns no significant * p< 0.05, **p<0.01, *** p<0.001.

Supplementary figure 5. Global assessments of CNOT3 mRNA targets

(**A**) Volcano plot showing down- and up-regulated and unchanged genes upon CNOT3 depletion. The three groups were subjected to analysis using supervised machine learning approach. (**B-C**) Fitted curves of train (B) vs. test (C) datasets used for gradient boosting. (**D-J**) Violin plots showing analysis of specific features (**D**) CDS-AG%; (**E**) 5' UTR length, (**F**) CDS length, (**G**) 3' UTR length and (**H**) 5' UTR GC%, (**I**) CDS GC% and (**J**) 5' UTR GC%. (**K**) Heatmap showing enrichment of amino acids encoding by genes upon CNOT3-KD upregulated; downregulated or unchanged. (**L-N**) Scatter plots showing highly reproducible detection of number of RiboSTAMP edited sites in three independent experiments of control (**L**) vs. KD-33 (**M**) and KD-37 (**N**) groups. (**O**) Metagene plot showing edits (≥ 0.5 confidence level) distribution along gene regions in control vs. CNOT3 depleted cells (KD-33 and KD-37).

.

Supplementary figure 6. **CNOT3 associates with translation machinery in AML**

(**A**) Representative images of CNOT3-Puro-Proximity Ligation Assay (PLA) and EEF1G-Puro-PLA performed in with puromycin untreated cells. (**B-C**) Quantitative summary of measurement of PLA foci intensity of (**B**) CNOT3-Puro-PLA (in figure 6D) and (**C**) EEF1G-Puro-PLA (in figure 6F). (**D**) Representative images of CNOT3-Puro-PLA performed in control (empty vector) vs. OV (overexpression of full length CNOT3) and overexpression of NOT box truncated CNOT3. (**E**) Quantitative summary of CNOT3-Puro-PLA in (**D**). (**F**) Representative images of CNOT3-EEF1E-PLA performed in control (empty vector) vs. OV (overexpression of full length CNOT3) and overexpression of NOT box truncated CNOT3. (**G**) Quantitative summary of CNOT3-EEF1E-PLA in (**F**). (**H**) Representative images of CNOT3-EEF1D-PLA performed in control (empty vector) vs. OV (overexpression of full length CNOT3) and overexpression of NOT box truncated CNOT3. (**I**)

Quantitative summary of CNOT3-EEF1D-PLA in (**H**). Each dot represents data obtained from one cell. All graphs show data as mean+/- s.e.m. Two-tailed Student's t test. * $p < 0.05$, *** $p < 0.001$. Each dot represents data obtained from one cell. All graphs show data as mean+/- s.e.m. Two-tailed Student's t test. * $p < 0.05$, *** $p < 0.001$.
