# Supplementary material for: Translation efficiency driven by CNOT3 subunit of the CCR4-NOT complex promotes leukemogenesis
Source: Nat Commun. 2024 Mar 15;15:2340. doi: 10.1038/s41467-024-46665-2 (PMC10943099; doi:10.1038/s41467-024-46665-2)
Supplement: Supplementary file 3 — Description of Additional Supplementary Files [file 41467_2024_46665_MOESM3_ESM.pdf]

Description of Additional Supplementary Files

File Name: Supplementary Data 1
Description: Information of Primary patient samples

File Name: Supplementary Data 2
Description: All sgRNA sequences - domain screening-Cnot3

File Name: Supplementary Data 3
Description: Hiseq sequencing results and analysis of CNOT3 sgRNA domain screening

File Name: Supplementary Data 4
Description: RNA-seq analysis of CNOT3 FL OV vs.control.

File Name: Supplementary Data 5
Description: RNA-seq analysis of CNOT3 truncated OV vs.control

File Name: Supplementary Data 6
Description: RNA-seq analysis of CNOT3 KD vs.control

File Name: Supplementary Data 7
Description: Ranked list of proteins in mass spectrometry analysis of CNOT3 KD vs. control

File Name: Supplementary Data 8
Description: Protemic analysis of CNOT3 KD vs. control

File Name: Supplementary Data 9
Description: Ranked list of genes in RNA-seq analysis of CNOT3 KD vs. control

File Name: Supplementary Data 10
Description: Differential EPKM upon CNOT3 KD

File Name: Supplementary Data 11
Description: Enrichr analysis of Hallmark pathways of genes with EPKM upregulated in CNOT3 KD

File Name: Supplementary Data 12
Description: Enrichr analysis of Hallmark pathways of genes with EPKM downregulated in CNOT3 KD

File Name: Supplementary Data 13
Description: List of proteins identified associating with CNOT3 via IP mass spectrometry

File Name: Supplementary Data 14
Description: Enrichr analysis of CNOT3 interacting protein networks
