# Supplementary material for: Translation efficiency driven by CNOT3 subunit of the CCR4-NOT complex promotes leukemogenesis
Source: Nat Commun. 2024 Mar 15;15:2340. doi: 10.1038/s41467-024-46665-2 (PMC10943099; doi:10.1038/s41467-024-46665-2)
Supplement: Supplementary file 18 — Reporting Summary [file 41467_2024_46665_MOESM18_ESM.pdf]

nature portfolio

Corresponding author(s): Ly P Vu

Last updated by author(s): Sep 15, 2023

Reporting Summary

Nature Portfolio wishes to improve the reproducibility of the work that we publish. This form provides structure for consistency and transparency in reporting. For further information on Nature Portfolio policies, see our Editorial Policies and the Editorial Policy Checklist.

Statistics

For all statistical analyses, confirm that the following items are present in the figure legend, table legend, main text, or Methods section.

n/a	Confirmed	
☐	☒	The exact sample size (*n*) for each experimental group/condition, given as a discrete number and unit of measurement
☐	☒	A statement on whether measurements were taken from distinct samples or whether the same sample was measured repeatedly
☐	☒	The statistical test(s) used AND whether they are one- or two-sided<br>*Only common tests should be described solely by name; describe more complex techniques in the Methods section.*
☒	☐	A description of all covariates tested
☒	☐	A description of any assumptions or corrections, such as tests of normality and adjustment for multiple comparisons
☐	☒	A full description of the statistical parameters including central tendency (e.g. means) or other basic estimates (e.g. regression coefficient) AND variation (e.g. standard deviation) or associated estimates of uncertainty (e.g. confidence intervals)
☐	☒	For null hypothesis testing, the test statistic (e.g. *F*, *t*, *r*) with confidence intervals, effect sizes, degrees of freedom and *P* value noted<br>*Give P values as exact values whenever suitable.*
☒	☐	For Bayesian analysis, information on the choice of priors and Markov chain Monte Carlo settings
☒	☐	For hierarchical and complex designs, identification of the appropriate level for tests and full reporting of outcomes
☐	☒	Estimates of effect sizes (e.g. Cohen's *d*, Pearson's *r*), indicating how they were calculated

Our web collection on statistics for biologists contains articles on many of the points above.

Software and code

Policy information about availability of computer code

Data collection	All software used for data collection is commercially available. Those include: Zeiss Zen microscopy software. For flow cytometry and FACS data collection BD Fortessa or Aria equipment with BD FACSDiva software v1.0 were used. For RNA-seq data collection, Illumina HiSeq platform was used.
Data analysis	Statistical analysis was performed using Prism Graphpad (https://www.graphpad.com/scientific-software/prism/).For microscopy image, analysis ImageJ Version 2.0.0-rc-65/1.51w was used. Flow Cytometry data was analyzed on FlowJo version 10.8.1. For RNA-seq analysis, Cutadapt (v1.18)was used to trim reads and RSEM (v1.3.1) to align to either the human (Ensembl v38). Differential expression analysis was carried out using DEseq2 v1.40.2 with lfcShrink (type = "apeglm") to account for lowly expressed mRNAs. Differential expression analysis, visualisations and statistics used R version 4.3.1Pathway analysis was performed using Gene Set Enrichment Analysis (GSEA) (https://www.gsea-msigdb.org/gsea/index.jsp) and Enrichr (https://maayanlab.cloud/Enrichr/)Code available https://github.com/JamesEttles/CNOT3. For Ribostamp -seq analysis, C-to-U edits are identified as C-A mutations and quantified using the SAILOR (v1.2.0) analysis pipeline and for differential transcriptomic gene expression DESeq2 (v1.2.10; default parameters) was performed, and for differential EPKM level comparison, limma (v 3.56.2) was performed.

For manuscripts utilizing custom algorithms or software that are central to the research but not yet described in published literature, software must be made available to editors and reviewers. We strongly encourage code deposition in a community repository (e.g. GitHub). See the Nature Portfolio guidelines for submitting code & software for further information.

Data

Policy information about availability of data

All manuscripts must include a data availability statement. This statement should provide the following information, where applicable:

- Accession codes, unique identifiers, or web links for publicly available datasets
- A description of any restrictions on data availability
- For clinical datasets or third party data, please ensure that the statement adheres to our policy

Raw and assembled sequencing data from this study have been deposited in NCBI's Gene Expression Omnibus (GEO) under accession BioProject PRJNA985375. Proteomic mass spectrometry data are available at Zenodo via https://zenodo.org/record/8350804

Research involving human participants, their data, or biological material

Policy information about studies with human participants or human data. See also policy information about sex, gender (identity/presentation), and sexual orientation and race, ethnicity and racism.

Reporting on sex and gender	Primary patient samples were used in the study. All information including sex/gender was included in supplemental table 1.
Reporting on race, ethnicity, or other socially relevant groupings	n/a
Population characteristics	n/a
Recruitment	n/a
Ethics oversight	All study is approved by the Research Ethic Board of University of British Columbia.

Note that full information on the approval of the study protocol must also be provided in the manuscript.

Field-specific reporting

Please select the one below that is the best fit for your research. If you are not sure, read the appropriate sections before making your selection.

☒ Life sciences ☐ Behavioural & social sciences ☐ Ecological, evolutionary & environmental sciences

For a reference copy of the document with all sections, see nature.com/documents/nr-reporting-summary-flat.pdf

Life sciences study design

All studies must disclose on these points even when the disclosure is negative.

Sample size	For the animal studies we analyzed at least 5 animals per group to provide at least 80% power to detect at least 10% difference between mean (with standard deviation equal or less than half of difference between mean) value of control (WT) vs. tested (KO) group at the 0.05 significance level. For in vitro experiments in leukemia cells, sequencing, proteomics, and western blot, we aimed for a number of at least 3 independent experiments per group to allow for statistical inference using Student's t test (two tailed unless otherwise stated).
Data exclusions	No data were excluded from analysis.
Replication	For each experiments, at least 3 independent biological replicates were performed. All replications validated for effecient depletion or overexpression of genes of interest were successful.
Randomization	We allocated recipient mice into different groups randomly in transplant in vivo experiments. Animals in all experiment groups are sex and age matched. No other randomization was performed in the study as randomization is not applicable in experimental design where there is no perceived bias in using same cell samples for experimental manipulations.
Blinding	The authors were not blinded. Validation of efficient depletion or overexpression of genes of interest - must be confirmed prior to experimental assessments.

Reporting for specific materials, systems and methods

We require information from authors about some types of materials, experimental systems and methods used in many studies. Here, indicate whether each material, system or method listed is relevant to your study. If you are not sure if a list item applies to your research, read the appropriate section before selecting a response.

Materials & experimental systems

n/a	Involved in the study
☐	☒ Antibodies
☐	☒ Eukaryotic cell lines
☒	☐ Palaeontology and archaeology
☐	☒ Animals and other organisms
☐	☒ Clinical data
☒	☐ Dual use research of concern
☒	☐ Plants

Methods

n/a	Involved in the study
☒	☐ ChIP-seq
☐	☒ Flow cytometry
☒	☐ MRI-based neuroimaging

Antibodies

Antibodies used

Target antigene an clone, vendor, catalog number, application (dilution)
CNOT3 clone 4B8, Abnova, H00004849-M01, immunoblot (1:1000), immunofluorescence (1:000)
c-MYC clone D84C12 -NEB, 5605S, immunoblot (1:1000), immunofluorescence (1:000)
p21 clone 21D1 -Cell Signaling Technology 2947S, immunoblot(1:1000]
ACTIN clone AC-15, Sigma Aldrich, A3854,immunoblot (1:5000)
Anti-mouse AF488: Invitrogene (A-21202), Immunoflourescence (1:500)
anti-mouse AF568: Invitrogene(A10037) Immunoflourescence (1:100)
anti-rabbit AF568: Invitrogene (A10042) Immunoflourescence (1:500)
anti-rabbit AF647: Invitrogene (A32795) Immunoflourescence (1:500)
Rabbit α-puromycin clone ARC58626 -Abclonal, A23031, immunoflourescence (1:50)
mouse α-EEF1G clone 3F11-1A10 - Novus; H00001937-M01, immunoflourescence (1:100)
Rabbit α-EEF1D Abcam ab85964, immunoflourescence (1:100)
APC-CD11b clone VIM12, Invitrogene CD11B05, flow analysis (1:200)
FITC-CD13 clone VM15, eBioscience/ThermoFisher 11-0138-42, flow analysis (1:200)
PE-CD14 clone 61D3, eBioscience/ThermoFisher, 12-0149-42, flow analysis (1:200)
AF700-hCD45 clone 2D1, Biolegend, 368514, flow analysis (1:200)
PE- hCD45 clone H130, eBioscience,12-0459-42, flow analysis (1:200)

Validation

All antibodies used were purchased from commercial vendors and have beehttps://www.cellsignal.com/products/primary-antibodies/c-myc-d84c12-rabbit-mab/5605?_requestid=1651037n validated by the vendors. Information on each antibody and their validated application is available at the manufacture's website, we also list the validation for each primary antibody below. We used only for validated applications.
CNOT3 clone 4B8, Abnova, H00004849-M01, immunoblot (1:1000), immunofluorescence (1:000)
https://www.abnova.com/products/products_detail.asp?catalog_id=H00004849-M01A
c-MYC clone D84C12 -NEB, 5605S, immunoblot (1:1000), immunofluorescence (1:000)
https://www.cellsignal.com/products/primary-antibodies/c-myc-d84c12-rabbit-mab/5605?_requestid=1651037
p21 clone 21D1 -Cell Signaling Technology 2947S, immunoblot(1:1000]
https://www.cellsignal.com/products/primary-antibodies/p21-waf1-cip1-12d1-rabbit-mab/2947
ACTIN clone AC-15, Sigma Aldrich, A3854,immunoblot (1:5000)
https://www.sigmaaldrich.com/VN/en/product/sigma/a5441
Anti-mouse AF488: Invitrogene (A-21202), Immunoflourescence (1:500)
https://www.thermofisher.com/antibody/product/Donkey-anti-Mouse-IgG-H-L-Highly-Cross-Adsorbed-Secondary-Antibody-Polyclonal/A-21202
anti-mouse AF568: Invitrogene(A10037) Immunoflourescence (1:100)
https://www.thermofisher.com/antibody/product/Donkey-anti-Mouse-IgG-H-L-Highly-Cross-Adsorbed-Secondary-Antibody-Polyclonal/A10037
anti-rabbit AF568: Invitrogene (A10042) Immunoflourescence (1:500)
https://www.thermofisher.com/antibody/product/Goat-anti-Rabbit-IgG-H-L-Cross-Adsorbed-Secondary-Antibody-Polyclonal/A-11011
anti-rabbit AF647: Invitrogene (A32795) Immunoflourescence (1:500)
https://www.thermofisher.com/antibody/product/Goat-anti-Rabbit-IgG-H-L-Highly-Cross-Adsorbed-Secondary-Antibody-Polyclonal/A-21245
Rabbit α-puromycin clone ARC58626 -Abclonal, A23031, immunoflourescence (1:50)
https://abclonal.com/catalog-antibodies/PuromycinRabbitmAb/A23031
mouse α-EEF1G clone 3F11-1A10 - Novus; H00001937-M01, immunoflourescence (1:100)
https://www.novusbio.com/products/eef1g-antibody-3f11-1a10_h00001937-m01
APC-CD11b clone VIM12, Invitrogene CD11B05, flow analysis (1:200)
https://www.thermofisher.com/antibody/product/CD11b-Antibody-clone-VIM12-Monoclonal/CD11B05
FITC-CD13 clone VM15, eBioscience/ThermoFisher 11-0138-42, flow analysis (1:200)
https://www.thermofisher.com/antibody/product/CD13-Antibody-clone-WM-15-WM15-Monoclonal/11-0138-42
PE-CD14 clone 61D3, eBioscience/ThermoFisher, 12-0149-42, flow analysis (1:200)
https://www.thermofisher.com/antibody/product/CD14-Antibody-clone-61D3-Monoclonal/12-0149-42
AF700-hCD45 clone 2D1, Biolegend, 368514, flow analysis (1:200)
https://www.biolegend.com/en-us/products/alexa-fluor-700-anti-human-cd45-antibody-12399
PE- hCD45 clone H130, eBioscience,12-0459-42, flow analysis (1:200)
https://www.thermofisher.com/antibody/product/CD45-Antibody-clone-HI30-Monoclonal/12-0459-42

Eukaryotic cell lines

Policy information about cell lines and Sex and Gender in Research

Cell line source(s)	All cell lines were previously purchased from ATCC and validated and tested negative for mycoplasma contamination.
Authentication	All leukemia cell lines including MOLM-13, NB4,OCI-AML3, HL-60, NOMO-1, MV4-11 were authenticated via STR profiling using Genetica cell line testing service. 293T cells were used to generate viruses were not authenticated.
Mycoplasma contamination	All cells used are tested and negative for mycoplasma contamination.
Commonly misidentified lines (See ICLAC register)	No commonly misidentified cell lines were used in the study.

Animals and other research organisms

Policy information about studies involving animals; ARRIVE guidelines recommended for reporting animal research, and Sex and Gender in Research

Laboratory animals	NSG (NOD-scid IL2Rgnull) and NRG-3GS (NOD.Rag1-/-;γcnull-IL3/GM/SF) 8–10-week-old female mice were used for in vivo transplantation. Animals were housed with the light/dark cycle 12/12; ambient temperature 20-26 degree C; 40-60% relative humidity.
Wild animals	No wild animals were used in this study.
Reporting on sex	Female mice were used as recipient mice for in vivo transplantation to optimize the engraftability.<br>Information of clinical specimens of primary patient samples were reported and included in supplemental table 1. Samples were selected to included both biological identified sex.
Field-collected samples	No field-collection was used in this study.
Ethics oversight	The study complies with all relevant biosafety, animal procedures and ethical regulations as approved by the University of British Columbia biosafety committee, animal care and use committee and human ethic board.

Note that full information on the approval of the study protocol must also be provided in the manuscript.

Clinical data

Policy information about clinical studies

All manuscripts should comply with the ICMJE guidelines for publication of clinical research and a completed CONSORT checklist must be included with all submissions.

Clinical trial registration	n/a
Study protocol	n/a
Data collection	n/a
Outcomes	n/a

Flow Cytometry

Plots

Confirm that:

☒ The axis labels state the marker and fluorochrome used (e.g. CD4-FITC).

☒ The axis scales are clearly visible. Include numbers along axes only for bottom left plot of group (a 'group' is an analysis of identical markers).

☒ All plots are contour plots with outliers or pseudocolor plots.

☒ A numerical value for number of cells or percentage (with statistics) is provided.

Methodology

Sample preparation	For CNOT3 intracellular flow cytometry analysis (IC flow), cells were washed with PBS and resuspended in 250 µl of 1.5% paraformaldehyde as fixative for 15 minutes in RT. After washing the pellet with PBS, 1ml cold methanol was added for 20 minutes to permeabilize the cells. The cells were washed and 5 µl of primary intracellular CNOT3 antibody and incubated for 30 minutes at 4°C. Cells were washed and stained with anti-mouse secondary antibody conjugated with fluorochrome AF488 (Invitrogen, A-21202), and incubated at room temperature for 30 minutes. The cells were resuspended in 200 µl of FACS

buffer, transferred in FACS tube and were analysed. For all surface marker staining, cells were incubated for 15-30 minutes and washed with PBS then resuspended in RPMI 2% FBS for flow cytometry.

Instrument	All flow cytometry samples were analyzed on a BD FACS LSR Fortessa instrument.
Software	Flow analysis were performed using FlowJo 10.8.1
Cell population abundance	For flow analysis, samples were run to acquire at least 10,000 events in gated cell population. Analyzed populations were back-gated to confirm purity.
Gating strategy	Cells were gated for live cells (FSC-A, SSC-A), then for singlets (single cells) with FSC-H and FSC-W and SSC-H and SSC-W. Cells were then gated according to analysis in specific experiment, described in figure legends. Comparisons between control and experimental cells were done on the same gating strategies.

☒ Tick this box to confirm that a figure exemplifying the gating strategy is provided in the Supplementary Information.
